# The Needs and Priorities for Government Grants for Traditional Korean Medicine: Comparing the Public and Traditional Korean Medicine Doctors

**DOI:** 10.1155/2016/2625079

**Published:** 2016-11-23

**Authors:** Min Kyung Hyun

**Affiliations:** Department of Preventive Medicine, College of Korean Medicine, Dongguk University, Gyeongju-si, Republic of Korea

## Abstract

This study was conducted to compare the need for research and development (R&D) of Traditional Korean Medicine (TKM) perceived by the public and Traditional Korean Medicine doctor (KMD) in. Survey data from 2462 people and KMD were utilized for this study. Overall, 25.10% of the public and 90.91% of KMD answered that government grants for TKM R&D were “extremely necessary.” The majority of respondents reported that grants were needed “for the advancement of science and technology in TKM” (public, 46.28%; KMD, 34.08%). Research regarding herbal medicine was the top priority of TKM R&D in both groups. However, “research facilities and training for researchers (27.85%)” was a close second priority of the public, but not KMD. Moreover, the public believed that safety from adverse effects and toxicity was a more important area of R&D in each discipline, but KMD did not find these to be important. The public and KMD generally agreed on the need for government grants for TKM R&D, but the public was more interested in safety than KMD. Therefore, government policy decision makers must consider opinions of both the public and KMD when planning government grants.

## 1. Introduction

Currently, many people are interested in traditional medicine (TM) worldwide [1]. In some countries, a high percentage of the population (20%–64%) use TM [2, 3], and 60% of South Koreans have had experience with Traditional Korean Medicine (TKM) [4]. However, TKM is often unable to maintain its traditional status because it is now required to demonstrate the safety, efficacy, effectiveness, and quality control of TKM products. The demand for scientific evidence related to TM is also increasing as evidence-based medicine is expanding in many countries [5–9]. Accordingly, promoting safe and effective use of TM through research is becoming more important [1, 10]. In South Korea, many traditional Korean medical doctors (KMD) have long utilized government grants for research and development (R&D) of TKM, but the R&D results have not been sufficient to meet the specific needs of the public. This is probably due to the difference in the priority of the public and KMD for TKM R&D. However, there is no related research about these priorities.

Therefore, this study was conducted to analyze and compare differences in the needs and priorities of the public and KMD using national survey data.

## 2. Methods

### 2.1. Data

**Figure 1:**
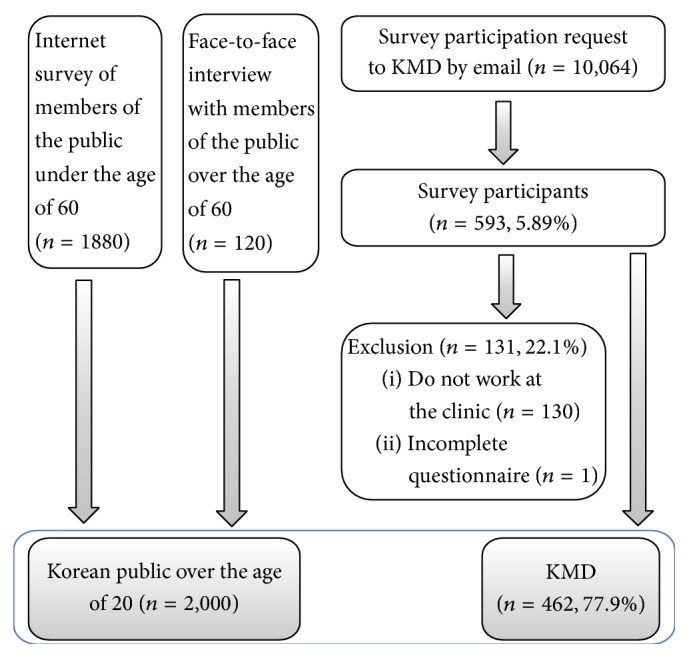
Structure of survey data.

A public survey was conducted to generate a national representative sample. The survey was conducted based on random emails (1880 adults under the age 60) and face-to-face interviews (120 adults over the age of 60) between December 2007 and January 2008 [4]. In addition, a KMD survey was conducted by emailing survey participants among KMD registered in the Association of Korean Medicine between September and October of 2008. After receiving written consent and answered survey questionnaires from 593 KMD, survey data from 131 KMD were excluded because they did not work in a clinical setting or did not complete the survey questionnaire (Figure 1).

The survey contents consisted of a demographic part and the need for government grants for TKM R&D including needs, reasons, and priorities among the disciplines of TKM R&D and area of each discipline.

This study was approved by the Institutional Review Board of the Dongguk University Gyeongju campus.

### 2.2. Statistical Analysis

Chi-squared tests were used to compare differences in outcomes between the public and KMD. A two-sided *p* value of <0.05 was considered to indicate statistical significance in this study. Statistical analyses were conducted using Stata/MP version 14 (StataCorp LP, College Station, Texas, USA).

## 3. Results

### 3.1. Study Population Characteristics

There were differences in all variables including sex, age, annual household income, and education, but regional variations were not significant. Most KMD were male (90%), aged 30–49 (90%), with an annual household income of over $120,000 (67%), and all had an education level of university or higher (100%). Conversely, 50% of the public were male, 51% were aged 30–49, 70% had an education level of university or higher, and 78% had an annual household income of $12,000 to $60,000 (Table 1).

### 3.2. The Need for Government Grants for TKM R&D

There were significant differences between the public and KMD regarding whether research grants were an “extreme necessity,” with 25.10% of the public and 90.91% of KMD responding to that positively. However, the majority of the public (81.35%) and KMD (98.7%) responded that it was a “necessity” or “extreme necessity,” while the public (1.75%) and KMD (0.22%) responded with “not necessary” or “not necessary at all.” Therefore, the public and KMD generally agreed on the need for government grants in TKM R&D (Table 2).

### 3.3. Reasons Why Government Grants Are Needed for TKM R&D

Respondents that felt government grants were a “necessity” or “extreme necessity” were asked to select a reason for this need. The majority of these respondents replied “for the advancement of science and technology in TKM,” while the second most common response was “for scientific interpretation of secret recipes” (25.88%) among public respondents and “for the improvement of TKM's competitiveness in the world market” (23.83%) among KMD. Among the public and KMD respondents, the third most common response was “to overcome the limitations of western medicine” (Table 3).

### 3.4. Priorities for TKM R&D

Herbal medicine was the first priority in both groups. However, 47.19% of the KMD placed a priority on herbal medicine R&D, while only 28.26% of the public prioritized this factor. The next greatest priority of the public was research facilities and training for researchers (27.85%), while for KMD it was “objectification of diagnostic technique.” KMD focused on technology itself, while the public prioritized basic investment in R&D (Table 4).

### 3.5. Priority Areas of R&D in Each Discipline TKM

Efficacy of herbal medicine, acupuncture/moxibustion/cupping, and medical devices was the first priority in both groups. Safety from adverse effects and toxicity was the more important research topic for the public but was not a significant factor for KMD. Conversely, development of new diagnostic devices (55.75% of KMD) and diagnostic assessment tools (53.80%) was high priorities for KMD, but not for the public (Table 5).

## 4. Discussion

To the best of our knowledge, this study is the first comparison of perception of the needs and priorities of government grants for TKM R&D of the public and KMD. The results showed significant differences between the public and KMD. Both groups agreed on the need for government grants in TKM R&D, but 3.6 times more KMD responded that there was “extreme necessity” than the public. Herbal medicine was the top priority of TKM in both groups, but 1.7 times more KMD responded positively to this question than the public.

To date, few studies have investigated research support for TKM [11–13], and we found no discussions based on the opinions of public and traditional medicine doctors about the needs and priorities for TM. An example of government grants for “research facilities and training for researcher” was that the National Center for Complementary and Alternative Medicine (NCCAM) had provided a research training and career development awards for complementary and alternative Medicine (CAM) [11], and the course of evidence-based medicine worked pretty well for CAM researcher [14].

In general, policy decision makers must consider the needs and priorities of both the public and experts before they determine whether to fund various research projects. However, in most cases, experts are brought in to advise government decision makers, while the opinions of the public are excluded. As a result, the priorities of the public, experts, and the government differ. For examples, the Ministry of Health & Welfare (MOHW) in the Republic of Korea invested about 60 million dollars (exchange rate: one US dollar equals 1,000 South Korean Won) for TKM R&D from 1999 to 2010. About 64% of the total funds were used to support R&D regarding herbal medicine and 15% was used to support research into TKM medical devices for diagnostic/treatment, while only 9% was utilized for research facilities and training for researchers. While no differences were found in the first priority of the three groups, the second most common use of MOHW grants was “medical device for diagnostic/treatment”; however, this was a very low priority for the public (9.4%) and KMD (4.98%). In other words, there were large gaps between the priority of government grants and priority disciplines between public and KMD groups. Moreover, the difference between the actual support provided by the government and public priority was larger than between that and KMD priority (Table 6). Moreover, in most of the total funds invested for efficacy research, there was almost no funding for safety research, including investigation of the adverse effects and toxicity that were considered to be important by the public.

Limitations regarding this study include the following: (a) the KMD survey data may not be fully representative because of the low response rate of KMD [15, 16]. However, in the case of KMD, methods other than email survey were impossible owing to limitations of the research budget and survey system used by the Association of Korean Medicine [17]. (b) Additionally, the results may not be appropriate for the current situation because the analytical data was collected in 2008. However, this was the best option given the lack of available data. Moreover, this study has great significance for the first comparison of differences in the needs and priorities of the public and KMD using two types of independent nationwide survey data.

Consequentially, government policy decision makers must consider the opinions of both the public and KMD when planning government grants. Safety research should be weighted more heavily than in the past.

## Figures and Tables

**Table 1:** Demographic characteristics of the respondents (*n* = 2,462).

	Public (*n* = 2,000)	KMD (*n* = 462)	*p*
Sex			<0.0001
Male	1000 (50%)	414 (90%)	
Female	1000 (50%)	48 (10%)	
Age (years)			<0.0001
20–29	425 (21%)	11 (2%)	
30–39	537 (27%)	250 (54%)	
40–49	489 (24%)	165 (36%)	
50–59	333 (17%)	31 (7%)	
60–69	216 (11%)	5 (1%)	
Annual household income^*∗*^		(*n* = 369)	<0.0001
<$12,000	126 (6%)	7 (2%)	
$12,000–<$60,000	1559 (78%)	46 (12%)	
$60,000–<$120,000	274 (14%)	69 (19%)	
≤$120,000	41 (2%)	247 (67%)	
Education			<0.0001
High school or less	606 (30%)	0 (0%)	
University or more	1,394 (70%)	462 (100%)	
Region			0.166
Capital	573 (29%)	142 (31%)	
Metropolitan	554 (28%)	108 (23%)	
And so forth	873 (44%)	212 (46%)	

KMD, Traditional Korean Medicine Doctors.

^*∗*^Exchange rate: one US dollar equals 1,000 South Korean Won.

**Table 2:** Perceived need for government grants for TKM R&D.

	Public (*n* = 2,000)	KMD (*n* = 462)	*p*
Extreme necessity	502 (25.10%)	420 (90.91%)	<0.0001
Necessity	1125 (56.25%)	36 (7.79%)	
Somewhat	338 (16.90%)	5 (1.08%)	
Not necessary	33 (1.65%)	0 (0.00%)	
Not necessary at all	2 (0.10%)	1 (0.22%)	

TKM, Traditional Korean Medicine; R&D, research and development.

**Table 3:** Perceived reasons government grants are needed for TKM R&D.

	Public (*n* = 1,627)^#^	KMD (*n* = 449)^#^	*p*
For the advancement of science and technology in TKM	753 (46.28%)	153 (34.08%)	<0.0001
For scientific interpretation of secret recipes	421 (25.88%)	49 (10.91%)	
To overcome the limitations of western medicine	239 (14.69%)	89 (19.82%)	
To improve TKM's competitiveness in the world market	107 (6.58%)	107 (23.83%)	
To generate evidence regarding TKM effectiveness	70 (4.30%)	35 (7.80%)	
Other	37 (2.27%)	16 (3.56%)	

# respondents of “extreme necessity” or “necessity” in Table 2.

**Table 4:** Priorities for TKM R&D.

	Public (*n* = 2,000)	KMD (*n* = 462)	*p*
Herbal medicine	565 (28.25%)	218 (47.19%)	<0.0001
Research facilities and training for researchers	557 (27.85%)	57 (12.34%)	
Objectification of diagnostic technique	369 (18.45%)	106 (22.94%)	
Acupuncture, moxibustion, cupping	315 (15.75%)	46 (9.96%)	
Medical device for diagnostic/treatment	188 (9.40%)	23 (4.98%)	
Other	6 (0.30%)	12 (2.60%)	

**Table 5:** Priority areas of R&D in each discipline of TKM.

	Public (*n* = 2,000)	KMD (*n* = 461)	*p*
*Herbal medicine*			
Efficacy	760 (38.00%)	153 (33.19%)	0.0176
Safety (adverse effect/toxicity)	501 (25.05%)	94 (20.39%)	
Quality control/standardization	330 (16.50%)	106 (22.99%)	
Convenient dosage form	303 (15.15%)	90 (19.52%)	
Combination therapy of TKM and WM	104 (5.20%)	8 (1.74%)	
Other	2 (0.10%)	10 (2.17%)	

*Acupuncture, Moxibustion, and Cupping*			
Efficacy	903 (45.15%)	211 (45.77%)	<0.0001
Safety (adverse effect/toxicity)	495 (24.75%)	40 (8.68%)	
Development of new technology	474 (23.70%)	159 (34.49%)	
Reduction of procedure related pain	125 (6.25%)	40 (8.68%)	
Other	3 (0.15%)	11 (2.39%)	

*Medical device for diagnostic/treatment*			
Efficacy	842 (42.10%)	138 (29.93%)	<0.0001
Safety (adverse effect/toxicity)	507 (25.35%)	19 (4.12%)	
Development of new diagnostic device	352 (17.60%)	257 (55.75%)	
Development of new treatment device	294 (14.70%)	42 (9.11%)	
Other	5 (0.25%)	5 (1.08%)	

*Research facilities and training for researcher*			
Researcher training strategy	935 (46.75%)	235 (50.98%)	0.649
Researcher utilization strategy	383 (19.15%)	75 (16.27%)	
Electronic medical record for TKM	363 (18.15%)	59 (12.80%)	
Utilization strategy of research facilities	169 (8.45%)	38 (8.24%)	
Support strategy of research facilities	146 (7.30%)	51 (11.06%)	
Other	4 (0.20%)	3 (0.65%)	

*Objectification of diagnostic technique*			
Comparative investigation of diagnostic procedures in WM and TKM	1123 (56.15%)	144 (31.24%)	<0.0001
Diagnostic assessment tools	507 (25.35%)	248 (53.80%)	
Collecting of traditional diagnostic techniques	368 (18.40%)	61 (13.23%)	
Other	2 (0.10%)	8 (1.74%)	

WM, western medicine.

**Table 6:** Comparison of priorities for TKM R&D among MOHW grants, the public, and KMD.

	MOHW^*∗*^	Public	KMD
Herbal medicine	1 (63.86%)	1 (28.25%)	1 (47.19%)
Medical device for diagnostic/treatment	2 (14.83%)	5 (9.40%)	5 (4.98%)
Research facilities and training for researcher	3 (8.96%)	2 (27.85%)	3 (12.34%)
Acupuncture, moxibustion, cupping	4 (7.79%)	4 (15.75%)	4 (9.96%)
Objectification of diagnostic technique	5 (3.74%)	3 (18.45%)	2 (22.94%)
Other	6 (0.82%)	6 (0.30%)	6 (2.60%)

^*∗*^MOHW grants from 1999 to 2010.

MOHW, Ministry of Health & Welfare (South Korea).
